# Mechanistic Insights and Advances of Bispecific T Cell Engaging Antibodies Therapy in Multiple Myeloma

**DOI:** 10.3390/medicina61122113

**Published:** 2025-11-27

**Authors:** Ting Fang Tang, Chin Sum Cheong, Chung Yeng Looi, Won Fen Wong, Gin Gin Gan

**Affiliations:** 1 Department of Medical Microbiology, Faculty of Medicine, Universiti Malaya, Kuala Lumpur 50603, Malaysia; tiffanytftang@gmail.com; 2 Department of Medicine, Faculty of Medicine, Universiti Malaya, Kuala Lumpur 50603, Malaysia; chinsum@ummc.edu.my; 3 School of Biosciences, Faculty of Health & Medical Sciences, Taylor’s University, Subang Jaya 47500, Malaysia; chungyeng.looi@taylors.edu.my; 4 Digital Health and Medical Advancement Impact Lab, Taylor’s University, Subang Jaya 47500, Malaysia

**Keywords:** bispecific antibodies, T cell engaging antibodies (TCEs), multiple myeloma, BiTEs, BCMA, Immunotherapy, cytokine release syndrome

## Abstract

Multiple myeloma (MM) is a clonal malignancy of terminally differentiated plasma cells characterized by bone marrow infiltration and excessive production of monoclonal immunoglobulins, leading to end-organ damage such as osteolytic bone lesions. Despite substantial therapeutic progress achieved with proteasome inhibitors, immunomodulatory drugs, and anti-CD38 monoclonal antibodies, multiple myeloma remains incurable, and outcomes for triple-class-refractory patients remain dismal, with median survival below one year. Bispecific T cell engaging antibodies (TCEs) have recently emerged as a promising immunotherapeutic approach capable of redirecting cytotoxic T cells to eliminate malignant plasma cells. These engineered antibodies simultaneously engage CD3 on T cells and a tumor-associated antigen such as B cell maturation antigen (BCMA), G protein-coupled receptor family C group 5 member D (GPRC5D), or Fc receptor homolog 5 (FcRH5), thereby forming an immune synapse that triggers T cell activation, cytokine secretion, and perforin–granzyme-mediated apoptosis of the targeted B cell. This review summarizes the molecular design, mechanism of action, and clinical development of TCEs in MM, encompassing early bi-specific T cell engagers (BiTE) constructs such as AMG 420 and next-generation IgG-like molecules including teclistamab. Pivotal clinical trials have demonstrated overall response rates between 43% and 73%, accompanied by durable remissions and manageable safety profiles. Future directions include earlier-line integration, synergistic combinations with immunomodulatory or costimulatory agents, and the development of trispecific formats to overcome antigen escape and T cell exhaustion. Collectively, TCEs represent a paradigm shift toward durable, immune-mediated disease control in multiple myeloma.

## 1. Introduction

Multiple myeloma is a malignant plasma cell disorder characterized by the clonal proliferation of abnormal plasma cells within the bone marrow, resulting in excessive production of monoclonal immunoglobulins and subsequent end-organ damage, including osteolytic bone lesions, anemia, renal impairment, and hypercalcemia [1]. Under normal physiological conditions, antigen-stimulated naïve B cells undergo somatic hypermutation and class-switch recombination within germinal centers before differentiating into long-lived plasma cells capable of secreting high-affinity antibodies. This process is tightly regulated by a network of transcription factors, most notably B lymphocyte–induced maturation protein 1 (BLIMP-1)–interferon regulatory factor 4 (IRF4)–X-box binding protein 1 (XBP1) triad [2], as well as other factors such as B cell lymphoma 6 protein (BCL6) and Runt-related transcription factor 1 (RUNX1), which coordinate plasma cell differentiation and antibody secretion [3,4].

Multiple myeloma arises when plasma cells acquire genetic and epigenetic alterations that enable them to escape normal immune surveillance and bone marrow microenvironmental regulation, leading to unchecked proliferation and accumulation within the marrow compartment [5,6]. Recurrent chromosomal translocations involving the immunoglobulin heavy chain (IgH) locus, such as *t*(11;14)(q13;q32), lead to overexpression of *Cyclin D1 (CCND1)* and represent one of the most frequent genetic lesions in multiple myeloma. Other copy number abnormalities, including gain of 1q21 and deletion of 17p13 affecting *TP53*, are associated with adverse prognosis. In parallel, mutations in genes regulating key signaling pathways, such as *Kirsten Rat Sarcoma Viral Oncogene Homolog (KRAS)* and *TNF Receptor–Associated Factor 3 (TRAF3)*, contribute to uncontrolled proliferation and immune evasion. Epigenetic reprogramming, including aberrant DNA methylation and dysregulation of histone-modifying enzymes, further amplifies oncogenic transcriptional programs and plasma cell plasticity, driving disease progression and therapeutic resistance [7,8]. The *t*(4;14)(p16;q32) translocation juxtaposes the *Multiple Myeloma SET domain (MMSET)* gene under the influence of the *IgH* enhancer, resulting in its aberrant overexpression. *MMSET* functions as a histone methyltransferase that catalyzes H3K36 dimethylation, leading to widespread chromatin remodeling, altered gene expression, and global epigenetic reprogramming that fuels myelomagenesis [9,10].

Multiple myeloma represents approximately 1–2% of all cancers and accounts for nearly 10% of hematologic malignancies worldwide, with incidence rising particularly among aging populations. Despite major therapeutic advances over the past two decades with proteasome inhibitors, immunomodulatory drugs (IMiDs), and monoclonal antibodies targeting CD38, multiple myeloma remains largely incurable, with most patients eventually experiencing disease relapse and drug resistance. Those refractory to all three major drug classes, defined as triple-class-refractory disease, face particularly poor outcomes, with a median overall survival of around 8 months [11,12]. In response to this clinical challenge, numerous ongoing studies are exploring next-generation immunotherapies, including bispecific T cell engaging antibodies (TCEs), chimeric antigen receptor-T (CAR-T) cell therapies, and antibody-drug conjugates, aimed at restoring immune surveillance and achieving durable remissions in patients [13,14,15]. Among these, TCEs have emerged as a transformative immunotherapeutic platform, capable of harnessing the cytotoxic potential of T lymphocytes to selectively eliminate malignant plasma cells. Understanding the underlying mechanism of action of bispecific T cell engagers is essential to appreciate their therapeutic potential and the rationale behind their clinical efficacy in multiple myeloma. The following sections provide an integrated overview of TCEs–based therapy in multiple myeloma, beginning with the molecular architecture and cytolytic pathways that mediate TCEs-induced plasma cell apoptosis. The discussion then traces the technological evolution of T cell-redirecting bispecific antibodies engineering from the early generation of bispecific T engagers (BiTEs) construct to next-generation IgG-like format, highlighting innovations that have enhanced stability and clinical applicability. Finally, key clinical milestones, therapeutic challenges, and emerging strategies are summarized to illustrate how TCEs are redefining the current and future treatment paradigms of multiple myeloma.

## 2. Mechanism of Action of Bispecific T Cell Engagers

**Figure 1:**
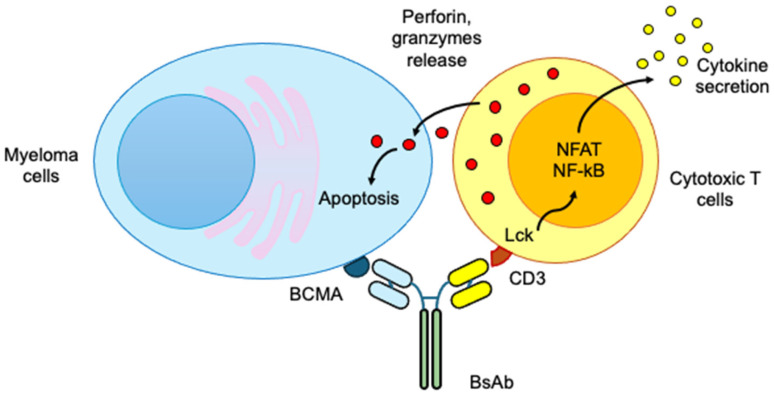
Mechanistic overview of TCE–mediated cytotoxicity in multiple myeloma. The schematic illustrates the immune mechanism triggered by bispecific antibodies that link cytotoxic T cells to malignant plasma cells. The TCE molecule contains two distinct antigen-binding domains, one specific for the CD3ε subunit on T cells and the other for a myeloma-associated surface antigen such as BCMA, GPRC5D, or FcRH5. Engagement of TCE or bispecific antibodies (BsAb) on CD3ε on T cell receptor complex leads to activation of intracellular signaling cascades involving Lck, ZAP70, and downstream transcription factors such as NFAT and NF-κB. These signals drive T cell activation and secretion of pro-inflammatory cytokines, including IFN-γ, TNF-α, and IL-2. The activated T cell releases cytolytic granules containing perforin and granzymes which causes apoptosis in target cell.

By design, TCEs are molecularly optimized to recruit and activate cytotoxic T cells in proximity to malignant plasma cells through dual antigen recognition. TCEs are recombinant antibody constructs designed to possess two antigen-binding domains with distinct specificities. One arm targets a tumor-associated antigen expressed on myeloma cells, such as B cell maturation antigen (BCMA), G-protein-coupled receptor class C group 5 member D (GPRC5D), or Fc receptor homolog 5 (FcRH5), while the other binds the CD3ε subunit of the T cell receptor (TCR) complex on cytotoxic T lymphocytes. This configuration physically bridges the T cell and the malignant plasma cell, enabling their close juxtaposition at the immune synapse (Figure 1). BCMA is a tumor necrosis factor receptor (TNFR) superfamily member, plays a critical role in plasma cell survival by binding a proliferation-inducing ligand (APRIL) and B cell activating factor (BAFF). Despite being the primary target of most first-generation bispecific, BCMA shedding by γ-secretase and antigen loss contribute to relapse. Therefore, GPRC5D and FcRH5 emerged as alternative targets due to their high expression on malignant plasma cells and minimal expression on vital tissues. GPRC5D shows restricted epithelial distribution, while FcRH5 is uniformly expressed across B cell lineage, making it an ideal candidate for pan–plasma cell targeting.

The engagement of the CD3 complex triggers synapse formation and phosphorylation of the immunoreceptor tyrosine-based activation motifs (ITAMs) on the CD3ζ chain, recruiting Src-family kinases such as lymphocyte-specific protein tyrosine kinase (Lck) and initiating downstream signaling cascades via zeta-chain-associated protein kinase 70 (ZAP70). These early events culminate in activation of the Nuclear factor of activated T cells (NFAT), Nuclear factor-κB (NF-κB) transcription factors, leading to proliferation, cytokine secretion, and cytolytic function. Once engaged by TCEs, T cells undergo rapid expansion, marked by upregulation of surface markers such as CD69 and CD25, and secrete a cascade of pro-inflammatory cytokines including interferon-gamma (IFN-γ), tumor necrosis factor-alpha (TNF-α), and interleukin 2 (IL-2). These cytokines amplify the local immune response by recruiting additional immune effectors, enhancing antigen presentation, and promoting the cytolytic capacity of neighbouring T cells. However, excessive cytokine production can lead to systemic inflammation known as cytokine release syndrome which typically manifests as fever and elevated inflammatory markers. Corticosteroid prophylaxis and IL-6 receptor blockade with tocilizumab are commonly employed to mitigate the severity of this adverse effect [16,17].

Following activation, cytotoxic T lymphocytes (CTLs) polarize their secretory machinery toward the tumor cell interface. Lytic granules containing perforin and granzymes are released into the synaptic cleft. Perforin forms transient pores in the target cell membrane, facilitating the entry of granzymes, which are serine proteases that activate caspase-dependent and caspase-independent apoptotic pathways. Granzymes cleave intracellular substrates, culminating in mitochondrial depolarization and DNA fragmentation that lead to tumor cell apoptosis [18,19].

However, it is important to note that despite robust activation through CD3 engagement, this interaction alone provides only the primary activation signal (signal 1) without delivering the essential co-stimulatory signal (signal 2). The absence of this secondary input can result in incomplete T cell activation, limited proliferation, or eventual functional exhaustion. Recognizing this limitation has driven the design of next-generation T cell engagers that integrate co-stimulatory domains such as CD28 or 4-1BB to enhance T cell persistence, sustain effector function, and improve overall antitumor efficacy [20,21].

## 3. Advancements from First- to Next-Generation Bispecific Antibodies in Multiple Myeloma

**Figure 2:**
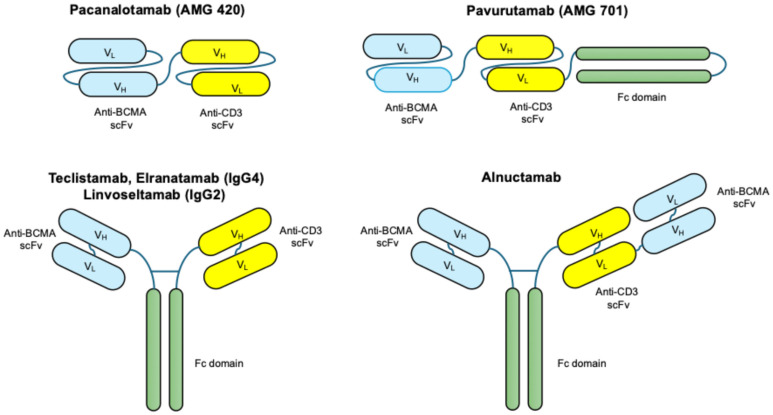
Structural design of TCEs used in multiple myeloma. The diagram illustrates the distinct molecular architectures of early generation BITEs, such as AMG420 and AMG701 (**left**), and new TCEs such as teclistamab (**right**). BiTEs consist of two single-chain variable fragments (scFvs) connected by a short linker, simultaneously binding CD3 on T cells and a tumor-associated antigen (e.g., CD19). Due to the absence of an Fc region, BiTEs have a short serum half-life, necessitating continuous intravenous infusion for sustained activity. Pavurutamab (AMG 701) incorporates an Fc-based half-life–extension module while retaining a BiTE-like CD3×BCMA targeting format, resulting in improved pharmacokinetic stability and allowing for intermittent dosing. In contrast, next-generation TCEs such as BCMA×CD3 antibodies (e.g., teclistamab, elranatamab and linvoseltamab) adopt a full-length IgG-like structure incorporating an Fc domain, which confers improved stability, extended half-life, and compatibility with subcutaneous or intermittent intravenous dosing. Alnuctamab represents a further evolution of this class, utilizing a 2+1 asymmetric IgG1 format that engages BCMA bivalently and CD3 monovalently, providing enhanced binding avidity to myeloma cells while modulating T cell activation strength.

The earliest bispecific T cell-redirecting antibodies, termed bi-specific T cell engagers (BiTEs), marked a pioneering step in harnessing immune effector cells for cancer therapy. The prototypical BiTE, blinatumomab (CD19×CD3), demonstrated remarkable efficacy in B cell acute lymphoblastic leukemia (B-ALL) by mediating potent T cell cytotoxicity through CD3 engagement and tumor antigen binding [22,23]. Structurally, BiTEs consist of two single-chain variable fragments (scFvs) linked by a short peptide, resulting in a compact, Fc-less molecule (Figure 2). The first-generation BiTE AMG 420 (BCMA×CD3), also named pacanalotamab, provided clinical proof of concept for T cell redirecting immunotherapy in multiple myeloma. In the phase I trial (NCT02514239), AMG 420 achieved an overall response rate (ORR) of 70% at the 400 µg/day dose in patients with relapsed/refractory multiple myeloma (RRMM), demonstrating potent anti-myeloma activity [24,25].

While first generation BiTEs allowed efficient synapse formation between the tumor and T cell, it was accompanied by significant pharmacokinetic limitations, including a very short serum half-life of approximately 1 to 2 hours, necessitating continuous intravenous infusion for clinical efficacy [26,27]. Moreover, the small molecular size increased renal clearance, while the lack of an Fc region prevented Fc-mediated recycling and stability. These pharmacologic constraints, combined with high rates of cytokine release syndrome and the need for continuous hospitalization, limited the broader applicability of early BiTEs to solid and plasma cell malignancies. To address the short half-life of AMG 420, pavurutamab (AMG 701) was engineered as a half-life–extended (HLE)-BiTE incorporating Fc- and albumin-binding modifications, allowing weekly or biweekly intermittent IV administration in phase I studies (NCT03287908) [28,29]. Nevertheless, the trial was prematurely discontinued due to strategic portfolio decisions by the sponsor to cease further development.

In recent years, next-generation bispecific antibodies were developed using full-length IgG scaffolds that retain an Fc region for enhanced stability and manufacturability. The Fc domain undergoes selective engineering to minimize Fcγ receptor and complement binding while preserving neonatal Fc receptor (FcRn)-mediated recycling, thereby extending serum half-life to several days or weeks. This innovation allows for intermittent intravenous or subcutaneous administration instead of continuous infusion. Examples include teclistamab and elranatamab, which maintain potent T cell-mediated cytotoxicity while enabling step-up dosing regimens to mitigate cytokine-related toxicities [30].

Teclistamab represents the first approved BCMA×CD3 full-length IgG4 bispecific antibody with an Fc-silenced backbone enabling FcRn-mediated recycling, providing a half-life of approximately 2 to 3 weeks and allowing subcutaneous step-up administration followed by weekly maintenance in the MajesTEC-1 trial (NCT03145181) [31,32]. Similarly, elranatamab, a humanized IgG2 BCMA-directed bispecific antibody with extended half-life properties, is administered subcutaneously using step-up priming followed by weekly and subsequently every-two-week dosing in responders in MagnetisMM-3 trial (NCT04649359) [33,34].

Additional next-generation candidates include linvoseltamab, a full-length IgG4 BCMA×CD3 bispecific antibody administered intravenously using a step-up schedule followed by weekly or every-two-week dosing in LINKER-MM1 trial (NCT03761108) [35,36]. In addition, alnuctamab, a structurally asymmetric 2+1 IgG1-based bispecific antibody with bivalent BCMA and monovalent CD3 binding that demonstrates prolonged IgG-like half-life with IV or subcutaneous administration in phase I/II CC-93269-MM-001 trials (NCT03486067) [37,38]. Collectively, these agents exemplify the rapid evolution of TCE engineering from short-acting BiTEs requiring continuous infusion to long-acting, Fc-bearing IgG-like antibodies compatible with intermittent and often subcutaneous dosing, thereby improving clinical feasibility, patient convenience, and long-term therapeutic potential. To facilitate comparison, we have summarized the core structural and pharmacological properties of the major BCMA- and GPRC5D-directed T cell engagers in multiple myeloma (Table 1).

## 4. Clinical Advances of Bispecific T Cell Engaging Antibodies in Multiple Myeloma

TCEs have established a new treatment paradigm in multiple myeloma by achieving rapid, deep, and durable responses in patients who have exhausted standard therapeutic classes. Both BCMA-directed agents (teclistamab, elranatamab, linvoseltamab) and non-BCMA antibodies (talquetamab and cevostamab), as discussed below offer valuable therapeutic alternatives in the management of relapsed refractory multiple myeloma (Table 2).

### 4.1. Teclistamab (Tecvayli™)

Teclistamab is the first-in-class BCMA×CD3 bispecific antibody approved by both the U.S. Food and Drug Administration (FDA) and the European Medicines Agency (EMA) for the treatment of relapsed and refractory multiple myeloma after at least four prior lines of therapy. In the pivotal phase I/II MajesTEC-1 study (NCT03145181), teclistamab demonstrated compelling efficacy in heavily pretreated, triple-class–refractory MM patients. The subcutaneous formulation yielded an overall response rate (ORR) of 63%, with complete response (CR) or better in 39% and minimal residual disease (MRD) negativity in 26% of evaluable patients [31,32,39]. Responses were durable, with a median duration of response (DOR) of 18 months and median progression-free survival (PFS) of 11.3 months, highlighting its capacity for sustained disease control even in advanced disease. Cytokine release syndrome (CRS) occurred in 72% of patients but was predominantly grade 1–2 and effectively mitigated by a step-up dosing schedule. Immune effector cell-associated neurotoxicity syndrome (ICANS) was infrequent (<3%), while infections and cytopenias represented the main non-cytokine toxicities. The step-up priming schedule and subcutaneous administration improved tolerability and outpatient feasibility. Beyond monotherapy, teclistamab is being evaluated in combination with other immunomodulatory and antibody agents such as daratumumab and lenalidomide in ongoing MajesTEC-4 trials, which aim to assess its integration into earlier lines of therapy [40].

### 4.2. Elranatamab (Elrexfio™)

Elranatamab is a humanized BCMA×CD3 bispecific antibody engineered with an extended half-life, enabling convenient weekly or biweekly subcutaneous administration. The phase II MagnetisMM-3 trial (NCT04649359) investigated elranatamab in patients with RRMM who had received ≥3 to 4 prior lines of therapy and were refractory to proteasome inhibitors, IMiDs, and anti-CD38 antibodies. The trial demonstrated an ORR of 61%, with very good partial response (VGPR) and better in 36% and MRD negativity in 31% of responders [33,34,41]. With extended follow-up, elranatamab achieved median DOR of approximately 17 months and median PFS between 12 to 15 months, depending on the dosing interval and cohort. The safety profile was manageable and consistent with the TCE class. CRS was reported in 58% of cases, primarily grade 1 to 2. ICANS occurred in 3% of patients, and most events were transient and reversible. The extended half-life supports sustained CD3 engagement and prolonged T cell activation while allowing flexibility in treatment scheduling. Its favorable safety and durability of response make elranatamab a strong contender among BCMA-directed TCEs, and it is now under evaluation in combination regimens and in earlier treatment lines, including maintenance settings after autologous stem cell transplantation.

### 4.3. Linvoseltamab (REGN5458)

Linvoseltamab represents a next-generation BCMA×CD3 bispecific antibody designed by Regeneron to optimize T cell engagement while minimizing cytokine toxicity. In the ongoing phase II LINKER-MM1 study (NCT03761108), linvoseltamab demonstrated robust anti-myeloma activity with an ORR of 71% among heavily pretreated RRMM patients who had received a median of five prior lines of therapy [35,42]. Responses were rapid, often occurring within the first treatment cycle, and maintained in a substantial proportion of patients beyond 12 months. The safety profile of linvoseltamab is favorable, with CRS reported in 46% of patients, nearly all grade 1 to 2, and severe events (<1%) being rare. Hematologic toxicities, including neutropenia and anemia, were manageable with supportive care. Ongoing clinical trials are investigating its integration into earlier treatment settings and potential synergy with IMiDs, anti-CD38 antibodies, and γ-secretase inhibitors to enhance BCMA expression and overcome resistance.

### 4.4. Alnuctamab (BMS-986349)

Alnuctamab is an investigational, next-generation BCMA×CD3 bispecific antibody that employs an asymmetric 2+1 full-length IgG1 architecture, enabling bivalent binding to BCMA and monovalent engagement of CD3 to optimize myeloma cell avidity while modulating T cell activation intensity. This structural design aims to enhance tumor-selective cytotoxicity, reduce excessive T cell stimulation, and improve safety compared to classical BiTE-like formats. In the first-in-human phase I study, CC-93269-MM-001 (NCT03486067), alnuctamab was evaluated in heavily pretreated RRMM patients, including those with triple-class refractory disease. Early data demonstrated high response rates, with ORR exceeding 80% at target-dose levels in some cohorts, including achievement of ≥VGPR and MRD-negative responses in a substantial proportion of responders [37,38,43]. However, despite encouraging clinical activity, the safety profile included notable rates of CRS, particularly during early dose-escalation, necessitating step-up priming strategies and optimized supportive-care protocols. Following strategic portfolio reprioritization by the developer, further clinical development of alnuctamab was discontinued despite strong biological rationale and antimyeloma activity, and the program is no longer enrolling new participants.

### 4.5. Talquetamab (Talvey™)

Talquetamab is the first bispecific antibody targeting the non-BCMA antigen G-protein-coupled receptor class C group 5 member D (GPRC5D), which is highly expressed on myeloma cells [44]. The phase I/II MonumenTAL-1 study (NCT03399799) evaluated talquetamab in patients with RRMM after ≥4 prior therapies, including those refractory to BCMA-directed agents. The study reported an ORR of 70% at 405 µg/kg and 64% at 800 µg/kg, with median DORs of 10.2 and 7.8 months, respectively [45,46]. The median PFS ranged from 7–8 months. Toxicities primarily reflected on-target, off-tumor effects related to GPRC5D expression in epithelial tissues, including dysgeusia, xerostomia, and nail changes. CRS occurred in around 78% of patients, predominantly grade 1 to 2, while infections and cytopenias were manageable with standard prophylactic measures.

### 4.6. Cevostamab (RG6160)

Cevostamab is a humanized bispecific antibody targeting Fc receptor homolog 5 (FcRH5), a pan-B cell surface protein consistently expressed on malignant plasma cells. In the Phase I GO39775 study (NCT03275103), the FcRH5×CD3 bispecific antibody cevostamab demonstrated meaningful clinical activity and manageable safety in heavily pretreated relapsed/refractory multiple myeloma [47]. Among 167 patients treated at the 160 mg every-3-weeks target dose, who had received a median of six prior therapies, the overall ORR was 43.1% and a median DOR of 10.4 months. The most common adverse event was CRS (74.3%; mostly grade 1 to 2, ≤2% grade ≥ 3), effectively mitigated by triple-step dosing. Other frequent toxicities included neutropenia (31%), anemia (23%), and infections (54%), with low incidence of severe neurotoxicity (13% ICANS, mainly grade 1 to 2). Overall, cevostamab achieved durable responses with a manageable safety profile.

## 5. Bispecific T Cell Engagers Versus Other Emerging Myeloma Immunotherapy Approaches

Bispecific TCEs have rapidly emerged as a transformative immunotherapeutic platform in RRMM. Their off-the-shelf availability, and immediate clinical deployability differentiate them from personalized cellular therapies such as CAR-T therapy, which require complex and time-intensive autologous production. Beyond TCEs, several other innovative immunotherapy classes are evolving toward broader integration in the myeloma treatment continuum (Table 3). CAR-T cell therapies offer the potential for highly durable remissions due to long-term cellular persistence and memory formation, although their use is limited by manufacturing logistics, treatment-related morbidity, and restricted access. Antibody-drug conjugates (ADCs) provide targeted cytotoxic payload delivery with standardized dosing but generally produce less durable responses and can induce characteristic toxicities such as corneal injury [48,49]. Cereblon E3 Ligase Modulators (CELMoDs), e.g., iberdomide, represent next-generation immune-modulating agents capable of enhancing T and NK cell fitness, potentially serving as synergistic partners for TCEs or CAR-T [50,51]. NK cell therapies, including engineered, memory-like, and engager-based constructs, aim to provide potent cytotoxicity with a reduced risk of cytokine-mediated toxicities [52,53]. Each modality offers distinct advantages in terms of potency, accessibility, toxicity, and logistical complexity, with TCEs occupying a strategic middle ground by providing greater efficacy than conventional antibodies or ADCs while remaining more accessible and rapidly deployable than autologous CAR-T cell therapy.

## 6. Challenges of TCEs Therapy in Multiple Myeloma

**Figure 3:**
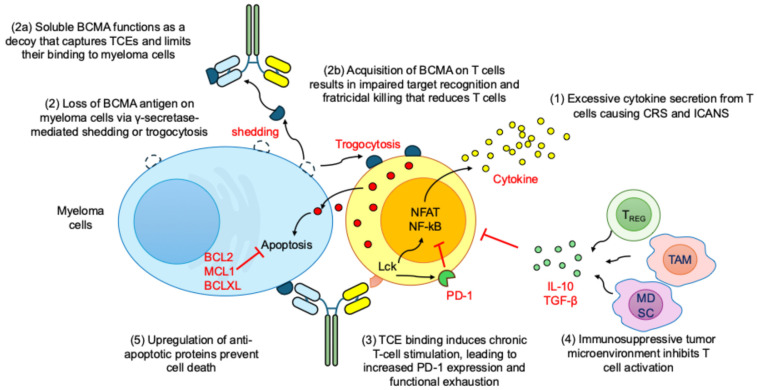
Challenges of TCEs treatment in multiple myeloma. The diagram illustrates multiple biological and microenvironmental barriers that limit TCE durability and therapeutic response. (1) TCE stimulation triggers robust immune synapse formation and excessive cytokine release from activated T cells, contributing to cytokine release syndrome (CRS) and immune effector cell-associated neurotoxicity syndrome (ICANS). (2) Loss or reduction in surface BCMA on myeloma cells occurs through γ-secretase-mediated shedding and trogocytosis, leading to diminished target density. (2a) Shed soluble BCMA acts as a decoy that captures circulating TCEs and reduces their engagement with tumor-expressed BCMA while (2b) acquisition of BCMA on T cell membranes results in impaired target recognition and fratricidal killing, further lowering functional T cell availability. (3) Persistent, high-intensity TCE signaling drives chronic T cell activation, resulting in increased PD-1 expression, transcriptional reprogramming, and functional exhaustion. (4) The immunosuppressive bone marrow niche, enriched with Tregs, MDSCs, TAMs and inhibitory cytokines (TGF-β, IL-10), attenuates T cell activation, expansion, and effector function. (5) Myeloma cells enhance survival signaling by upregulating anti-apoptotic molecules, including BCL-2, MCL-1, and BCL-xL, thereby resisting TCE-mediated cytotoxic apoptosis.

Despite the remarkable clinical efficacy of TCEs in RRMM, several challenges limit their broader application and long-term effectiveness [54,55]. These mechanisms highlight the need for next-generation TCEs that integrate multi-antigen targeting, optimized pharmacokinetics, enhancement of T cell fitness, and microenvironment modulation (Figure 3). Continued development of rational combination approaches and biomarker-guided treatment strategies will be critical for achieving more durable and sustained responses in multiple myeloma.

### 6.1. Adverse Effects of TCEs

The most common and clinically significant adverse events are CRS, cytopenias, and infection-related complications, most of which are manageable with step-up dosing, corticosteroids, interleukin 6 blockade, and prophylactic immunoglobulin therapy. Cytokine release syndrome is commonly seen in immunotherapy, which results from rapid T cell activation and cytokine overproduction, and it necessitates careful monitoring and supportive management. ICANS is infrequent but remains a potential concern due to excessive cytokine signaling within the central nervous system. Cytopenias, including neutropenia and lymphopenia, are frequent and contribute to increased susceptibility to bacterial, viral, and fungal infections. In addition, prolonged plasma cell depletion can result in hypogammaglobulinemia, predisposing patients to recurrent infections. Routine monitoring of immunoglobulin levels and intravenous immunoglobulin (IVIG) supplementation are recommended preventive measures which have been shown to abrogate most of the infection risk in patients [56]. Opportunistic infections, particularly herpes zoster reactivation and respiratory tract infections, are also common, emphasizing the need for antiviral prophylaxis and vaccination strategies.

### 6.2. Antigen Loss and Trogocytosis-Mediated Antigen Stripping

Antigen loss or downregulation is a major mechanism limiting the durability of BCMA-directed TCEs. A well-characterized example is BCMA shedding mediated by γ-secretase, which reduces membrane-bound BCMA and generates soluble BCMA that acts as a decoy for antibody binding. This reduces the density of targetable antigen on myeloma cells and interferes with effective synapse formation. To counter this problem, several approaches are being explored, including the use of γ-secretase inhibitors to prevent antigen shedding, the development of dual- or multi-antigen targeting TCEs such as BCMA×GPRC5D to minimize reliance on a single target [57,58].

Trogocytosis is a process in which immune cells extract and acquire fragments of the plasma membrane and surface proteins from other cells during direct cell-to-cell contact [59]. Trogocytosis is increasingly recognized as an additional form of antigen depletion in which T cells actively extract BCMA molecules from the surface of myeloma cells and acquire them on their own membranes [60,61]. This process reduces antigen availability on tumor cells and can contribute to T cell dysfunction or fratricidal interactions.

### 6.3. T Cell Exhaustion

Heavily pretreated myeloma patients often exhibit senescent, terminally differentiated, metabolically exhausted, or lymphopenic T cell pools, resulting in insufficient proliferative reserve following TCE activation. T cell exhaustion is characterized by the upregulation of inhibitory receptors such as Programmed cell death protein 1 (PD-1), lymphocyte-activation gene 3 (LAG-3), T cell immunoreceptor with Ig and ITIM domains (TIGIT), and T cell immunoglobulin and mucin-domain containing 3 (TIM-3); and a corresponding decline in proliferative capacity and cytotoxic function [62]. This exhausted phenotype reduces the persistence and effectiveness of TCE-redirected T cells over time [63,64].

### 6.4. Immunosuppressive Bone Marrow Microenvironment

The myeloma bone marrow microenvironment exerts substantial immunosuppressive pressure that limits TCE efficacy. Elevated numbers of regulatory T (T_REG_) cells, Myeloid-derived suppressor cells (MDSCs), tumor-associated macrophages (TAMs), and the presence of inhibitory cytokines such as transforming growth factor-β (TGF-β) and interleukin 10 (IL-10) collectively suppress T cell activation and persistence [65,66,67]. To counter these effects, several microenvironment-modifying strategies are under investigation, including combining TCEs with IMiDs or proteasome inhibitors to enhance immune activation, applying therapies that target or reprogram suppressive myeloid populations, and blocking pathways such as TGF-β or adenosine signaling that contribute to local immune suppression.

### 6.5. Altered Apoptotic Signaling in Myeloma Cells

Some myeloma cells resist immune-mediated cytotoxicity through intrinsic mechanisms involving the upregulation of anti-apoptotic proteins such as B cell lymphoma 2 (BCL-2), myeloid cell leukemia 1 (MCL-1), and B cell lymphoma-extra-large (BCL-XL). This shifts the balance toward survival even when effective T cell synapse formation and cytotoxic granule release occur [68,69]. Combining TCEs with apoptosis-sensitizing agents, such as Bcl-2 homology 3 (BH3) mimetics or proteasome inhibitors, is being explored as a way to lower the apoptotic threshold and enhance the effectiveness of T cell–mediated killing.

## 7. Predictive Biomarkers and Patient Selection for TCE Therapy

The identification of predictive biomarkers is increasingly recognized as a critical component in optimizing patient selection, maximizing therapeutic benefit, and minimizing immune-related toxicity associated with TCE therapy. As response to TCEs is dependent on both target expression on myeloma cells and the functional status of host T cells, biomarker development requires an integrated assessment of tumor-intrinsic, immune-intrinsic, and therapy-related determinants. One of the most extensively studied biomarkers is target antigen density, such as BCMA surface expression, which has been associated with TCE responsiveness. Low or heterogeneous antigen expression, particularly in heavily pretreated disease or after antigen-directed therapies, may reduce synaptic engagement and promote early relapse (Table 4).

In parallel, immune fitness biomarkers, including baseline T cell counts, and expression of exhaustion markers such as PD-1, are emerging as determinants of response quality and depth. Patients with reduced T cell reserve or predominance of terminally exhausted phenotypes may experience attenuated responses or shortened remission durations [70,71]. Similarly, soluble BCMA levels serve as a surrogate for antigen shedding and disease burden and may influence therapeutic efficacy by sequestering BCMA-directed agents [72].

The impact of prior therapies is another key consideration, as exposure to alkylating agents, high-dose corticosteroids, proteasome inhibitors, IMiDs, or prior cellular therapy may modify antigen expression, immune cell fitness, and microenvironmental immunosuppression. For example, post-CAR-T relapses frequently exhibit reduced antigen expression or immunologic exhaustion, which may impair response to subsequent TCE therapy and warrant alternative antigen targeting strategies [73,74]. Collectively, these biomarkers highlight that optimal patient selection is critical for maximizing TCE efficacy, minimizing toxicity, and guiding personalized treatment strategies in multiple myeloma.

## 8. Future Directions

TCEs therapy represents a rapidly advancing field, with continuous innovations in antibody engineering aimed at enhancing therapeutic efficacy while minimizing treatment-related toxicity [75,76]. A major engineering advancement involves the development of HLE-BiTE, achieved through Fc engineering, albumin-binding domains, or modified hinge regions that prolong systemic exposure and permit less frequent dosing schedules. Agents such as pavurutamab (AMG 701) and alnuctamab exemplify this approach, enabling intermittent rather than continuous or weekly dosing. At the same time, several next-generation constructs incorporate attenuated CD3-binding domains or stepwise activation mechanisms to reduce the risk and severity of CRS. These include low-affinity CD3 variants, Fc modifications to modulate T cell activation kinetics, and molecules designed for conditional activation within the tumor microenvironment, all of which aim to preserve antitumor potency while minimizing systemic toxicity.

Building upon the clinical success of first-generation BCMA-targeting agents such as teclistamab and elranatamab, current research is directed toward overcoming resistance, diversifying antigen targets, and optimizing treatment sequencing. A key innovation lies in the design of dual- and trispecific antibodies that engage multiple tumor antigens (e.g., BCMA/GPRC5D/CD3 or BCMA/CD3/CD28), thereby mitigating antigen escape and incorporating intrinsic co-stimulatory signaling to sustain T cell activation [77,78,79,80]. The integration of costimulatory domains such as CD28 or 4-1BB into next-generation constructs further enhances T cell persistence and cytolytic potency [21]. Preclinical studies have demonstrated that these dual-signaling designs not only prolong effector T cell survival but also mitigate exhaustion, collectively leading to more durable and effective antitumor responses [79,80,81].

Addressing antigen loss remains a central priority. In this context, pharmacologic inhibition of γ-secretase has emerged as a promising approach to prevent BCMA shedding and enhance therapeutic efficacy [82,83]. γ-secretase mediates the proteolytic cleavage of membrane-bound BCMA, generating soluble BCMA that reduces surface antigen density and impairs antibody binding. By blocking this enzymatic activity, γ-secretase inhibitors restore and stabilize BCMA expression on malignant plasma cells, thereby improving target accessibility and potentiating the cytolytic function of BCMA-directed T cell engagers.

In addition, ongoing clinical trials are actively exploring optimized treatment sequencing and combination regimens to enhance response durability [84,85]. The MajesTEC-4 trial (NCT05083169) is evaluating teclistamab plus daratumumab with lenalidomide in frontline maintenance settings, with primary endpoints expected in 2027 [40]. The TRIMM-2 study (NCT04108195), investigating teclistamab combined with daratumumab in patients with prior therapy exposure, has shown encouraging synergistic immune engagement, with additional follow-up analyses anticipated over the next 1 to 2 years [84]. Parallel efforts include MonumenTAL-3 (NCT05455320) and MagnetisMM-7 (NCT05317416), which examine talquetamab- and elranatamab-based regimens in earlier treatment settings and as maintenance therapy to prolong MRD negativity [86,87]. These pivotal results are projected to mature between 2025 and 2028, depending on accrual and follow-up. Collectively, these strategies signal a shift from TCEs as salvage therapy toward earlier-line integration to achieve deeper and longer-lasting responses.

## 9. Conclusions

TCEs represent a paradigm shift in MM therapy, offering deep and durable responses in patients with limited options after exposure to proteasome inhibitors, IMiDs, and anti-CD38 monoclonal antibodies. The success of BCMA-, GPRC5D-, and FcRH5-directed TCEs demonstrates the versatility of T cell-redirecting strategies. As ongoing studies continue to refine dosing, mitigate toxicity, and explore synergistic combinations, TCEs are anticipated to become integral components of both relapsed/refractory and frontline MM management. With multiple agents now FDA-approved and several others in late-phase development, TCEs are on the path to becoming foundational components of frontline and maintenance therapy for multiple myeloma. The combination of improved molecular design, optimized dosing, and integration with existing immunotherapies is likely to yield deeper, longer-lasting remissions and ultimately redefine long-term disease control. The next generation of multi-specific constructs and rational immune-oncology combinations hold the potential to transform multiple myeloma into a chronic, controllable disease.

## Figures and Tables

**Table 1:** Structure and Half-life of Bispecific Antibodies using BCMA and CD3 in Multiple Myeloma. IV: intraveneous; SC: subcutaneous; scFv: single-chain variable fragment.

Agent	Manufacturer (Headquarter City, Country)	Structure	Fc Domain	Half-Life	Route & Dosing Schedule
Pacanalotamab (AMG 420)	Amgen—Thousand Oaks, CA, USA	Tandem scFv–linker–scFv	No Fc	~2 h	Continuous IV infusion
Pavurutamab (AMG 701)	Amgen—Thousand Oaks, CA, USA	HLE-BiTE	Engineered Fc /albumin-binding	Days	Intermittent IV weekly/biweekly
Teclistamab (Tecvayli^®^)	Janssen/Johnson & Johnson—Titusville, NJ, USA	Full-length IgG4	Fc with effector function disabled	~2–3 weeks	SC (step-up) → weekly
Linvoseltamab (REGN5458)	Regeneron Pharmaceuticals—Tarrytown, NY, USA	Full-length IgG4	Fc with effector function disabled	~2–3 weeks	IV (step-up) → weekly/biweekly
Elranatamab (Elrexfio™)	Pfizer—New York, NY USA	Full-length IgG2	Fc with effector function disabled	~21–30 days	SC (step-up) → weekly → biweekly
Alnuctamab (BMS-986349)	Bristol Myers Squibb (BMS)—New York, NY, USA	Asymmetric 2+1 IgG-like	Fc with effector function disabled	Week-range	IV or SC (step-up) → weekly/biweekly

**Table 2:** Clinical Advances of Bispecific Antibodies in Multiple Myeloma.

Agent (Target)	Manufacturer	Key Trial	Population	ORR (%)	Median PFS (Months)
Teclistamab (BCMA×CD3)	Janssen/Johnson & Johnson—Titusville, NJ, USA	MajesTEC-1	≥5 prior lines, triple-class refractory	63	11.3
Elranatamab (BCMA×CD3)	Pfizer—New York, NY, USA	MagnetisMM-3	≥3 prior lines, prior BCMA-naïve	61	12–15
Linvoseltamab (BCMA×CD3)	Regeneron Pharmaceuticals—Tarrytown, NY, USA	LINKER-MM1	RRMM, median 5 prior lines	71	–
Talquetamab (GPRC5D×CD3)	Janssen/Johnson & Johnson—Titusville, NJ, USA	MonumenTAL-1	Heavily pretreated, post-BCMA allowed	73	7–8
Cevostamab (FcRH5×CD3)	Genentech/Roche (South San Francisco, CA, USA/Basel, Switzerland)	GO39775	RRMM, including prior BCMA exposure	56	8–9
Alnuctamab (BCMA×CD3)	Bristol Myers Squibb (BMS)—New York, NY, USA	CC-93269-MM-001	RRMM, heavily pretreated; ≥3–4 prior lines	~80 *	–

* ORR varied by dose level, reaching 80% at higher dose.

**Table 3:** Comparison of TCEs With Emerging Immunotherapy Modalities in Multiple Myeloma. GVHD: Graft versus host disease.

Modality	Format	Durability of Response	Key Toxicities	Cost	Facility
TCEs	Off-the-shelf bispecific antibodies	Variable; relapse possible due to antigen escape and T cell exhaustion	CRS/ICANS, cytopenias, infections	High	Immunotherapy trained centers
CAR-T Cell Therapy	Autologous genetically engineered T cells	Often long-lasting; potential for durable remission	CRS/ICANS, prolonged cytopenias, infections	Very high	Advanced cellular-therapy centers
ADC Therapy	Target-directed antibody–drug conjugate	Limited; typically transient disease control	Ocular toxicity, cytopenias, fatigue	Moderate–high	Standard oncology infusion units
CELMoDs	Oral small-molecule immune modulators	Moderate; often enhanced in combination	Cytopenias, rash, thrombosis	Moderate	Widely accessible outpatient settings
NK Cell Therapies	Allogeneic or engineered NK cell products	Under clinical investigation	Low CRS incidence	Very high	Advanced cellular-therapy centers

**Table 4:** Biomarkers Associated with TCEs Response, Toxicity, and Patient Selection.

Biomarker Category	Clinical Relevance
**Tumor-intrinsic factors**	
Cell-surface antigen level (BCMA)	Predicts likelihood of response and may inform optimal TCE selection.
Antigen depletion	Associated with resistance; supports development of multi-antigen or sequential TCE strategies.
Soluble BCMA concentration	Reflects tumor burden and antigen shedding; may reduce effective target engagement.
**Immune-intrinsic factors**	
Low CD4:CD8 T cell ratio	Predictive of suboptimal TCE responsiveness due to impaired helper-driven cytotoxic support.
T cell exhaustion markers (PD-1)	Associated with reduced proliferative capacity and weakened cytotoxic function.
**Microenvironmental factors**	
Abundant Tregs, MDSCs, TAMs	Indicates an immunosuppressive niche that limits T cell activation and persistence.
Immunosuppressive cytokines (TGF-β, IL-10)	Predict immune suppression; may justify concurrent modulation strategies.
Circulating cytokines (e.g., IL-6)	Correlate with CRS severity and toxicity risk, informing monitoring and prophylaxis.
Transcriptomic signatures	Predict T cell functional capacity and response durability.
**Treatment history-related factors**	
Prior CAR-T therapy	May alter antigen density, T cell resilience, or microenvironmental composition.
Exposure to IMiDs, PIs, steroids	Can modulate immune activation state, T cell quality, and downstream response.

## Data Availability

Not applicable.

## Abbreviations

The following abbreviations are used in this manuscript:

ADCs	Antibody-Drug Conjugates
APRIL	A Proliferation-Inducing Ligand
BAFF	B cell Activating Factor
B-ALL	B cell Acute Lymphoblastic Leukemia
BCMA	B cell Maturation Antigen
BCL-2	B cell Lymphoma 2
BCL-XL	B cell Lymphoma-Extra-Large
BCL6	B cell Lymphoma 6 Protein
BH3	Bcl-2 Homology 3
BiTE	Bispecific T Cell Engager
CAR-T	Chimeric Antigen Receptor T Cell
CELMoDs	Cereblon E3 Ligase Modulators
CR	Complete Response
CRS	Cytokine Release Syndrome
CTL	Cytotoxic T Lymphocyte
DOR	Duration of Response
EMA	European Medicines Agency
FDA	U.S. Food and Drug Administration
FcRH5	Fc Receptor Homolog 5
FcRn	Neonatal Fc Receptor
GPRC5D	G Protein-Coupled Receptor Family C Group 5 Member D
HLE-BiTE	Half-Life–Extended-BiTE
ICANS	Immune Effector Cell-Associated Neurotoxicity Syndrome
IFN-γ	Interferon-Gamma
IgG	Immunoglobulin G
IL-2	Interleukin 2
IL-6	Interleukin 6
IL-10	Interleukin 10
IMiD	Immunomodulatory Drug
IRF4	Interferon Regulatory Factor 4
ITAM	Immunoreceptor Tyrosine-Based Activation Motif
IVIG	Intravenous Immunoglobulin
LAG-3	Lymphocyte-Activation Gene 3
Lck	Lymphocyte-Specific Protein Tyrosine Kinase
MCL-1	Myeloid Cell Leukemia 1
MDSC	Myeloid-Derived Suppressor Cell
MM	Multiple Myeloma
MRD	Minimal Residual Disease
NFAT	Nuclear Factor of Activated T Cells
NF-κB	Nuclear Factor Kappa-Light-Chain-Enhancer of Activated B Cells
ORR	Overall Response Rate
PD-1	Programmed Cell Death Protein 1
PFS	Progression-Free Survival
T_REG_	Regulatory T
RUNX1	Runt-Related Transcription Factor 1
scFv	Single-Chain Variable Fragment
TAM	Tumor-Associated Macrophages
TCE	T Cell Engaging Antibody
TCR	T Cell Receptor
TGF-β	Transforming Growth Factor Beta
TIGIT	T Cell Immunoreceptor with Ig and ITIM Domains
TIM-3	T Cell Immunoglobulin and Mucin-Domain Containing 3
TNF-α	Tumor Necrosis Factor Alpha
TNFR	Tumor Necrosis Factor Receptor
VGPR	Very Good Partial Response
XBP1	X-Box Binding Protein 1
ZAP70	Zeta-Chain–Associated Protein Kinase 70
